# Delayed discharge is associated with higher complement C3 levels and a longer nucleic acid-negative conversion time in patients with COVID-19

**DOI:** 10.1038/s41598-021-81010-3

**Published:** 2021-01-13

**Authors:** Peihuang Lin, Wenhuang Chen, Hongbo Huang, Yijian Lin, Maosheng Cai, Dongheng Lin, Hehui Cai, Zhijun Su, Xibin Zhuang, Xueping Yu

**Affiliations:** 1 Department of Basic Medicine, Quanzhou Medical College, Quanzhou, China; 2 Department of Infectious Diseases, The First Hospital of Quanzhou Affiliated to Fujian Medical University, No. 250 East Street, Licheng District, Quanzhou, 362000 Fujian China; 3 Department of Respiratory Diseases, The First Hospital of Quanzhou Affiliated to Fujian Medical University, No. 250 East Street, Licheng District, Quanzhou, 362000 Fujian China; 4 Department of Respiratory Diseases, Shishi City General Hospital, Quanzhou, China; 5 Department of Respiratory Diseases, Anxi County Hospital, Quanzhou, China; 6 Department of Clinical Laboratory, The First Hospital of Quanzhou Affiliated to Fujian Medical University, Quanzhou, China

**Keywords:** Diseases, Health care, Risk factors

## Abstract

To determine factors associated with delayed discharge of hospitalized patients with coronavirus disease (COVID-19). This retrospective cohort study included 47 patients with COVID-19 admitted to three hospitals in Quanzhou City, Fujian Province, China, between January 21, 2020 and March 6, 2020. Univariate and multivariate logistic regression analyses were conducted to identify factors associated with delayed discharge. The median length of hospital stay was 22 days. Patients in the delayed discharge group (length of hospital stay ≥ 21 days, n = 27) were more likely to have diarrhea, anorexia, decreased white blood cell counts, increased complement C3 and C-reactive protein levels, air bronchograms, undergo thymalfasin treatment, and take significantly longer to convert to a severe acute respiratory syndrome coronavirus (SARS-CoV-2) RNA-negative status than those in the control group (length of hospital stay, < 21 days; n = 20). In multivariate logistic regression analysis, the time to SARS-CoV-2 RNA-negative conversion (odds ratio [OR]: 1.48, 95% confidence interval [CI] 1.09–2.04, *P* = 0.01) and complement C3 levels (OR 1.14 95% CI 1.02–1.27, *P* = 0.03) were the only risk factors independently associated with delayed discharge from the hospital. Dynamic monitoring of complement C3 and SARS-CoV-2 RNA levels is useful for predicting delayed discharge of patients.

## Introduction

Since December 2019, a new form of coronavirus pneumonia, coronavirus disease (COVID-19), caused by severe acute respiratory syndrome coronavirus (SARS-CoV-2), has caused a pandemic^1–4^. The common symptoms of COVID-19 include fever, lassitude, and dry cough. Similarly, some patients experience abdominal pain, diarrhea, nausea and vomiting, loss of appetite, and other gastrointestinal symptoms. A meta-analysis and systematic evaluation^5^ showed that 17.6% of patients with COVID-19 developed gastrointestinal symptoms, the most common of which were anorexia (26.8%), diarrhea (12.5%), nausea and vomiting (10.2%), and abdominal pain (9.2%). However, the timing and severity of gastrointestinal symptoms vary among different populations^6^. Moreover, the lymphocyte count is often reduced, and lung imaging usually shows ground-glass opacities in both lungs. The lesions are distributed mainly in the peripheral and subpleural regions of the lungs. Approximately 10% of those infected develop severe or critical disease, and case-fatality rates are high^7–11^. The large number of patients requiring hospitalization may exceed hospital capacity, and in many countries and regions, it is difficult to increase hospital capacity to meet the demand in a short period^12,13^. Therefore, increasing patient turnover and determining factors affecting the length of hospital stay may help relieve pressure on hospital admissions; however, there are no published studies on risk factors for delayed hospital discharge. Therefore, this study aimed to determine the clinical characteristics and duration of SARS-CoV-2 RNA shedding in patients confirmed to have COVID-19 and factors associated with delayed discharge from hospital.

## Methods

### Ethics declarations

This study was approved by the Ethics Committee of Fujian Provincial Center for Disease Control and Prevention (Min Ji Kong Lun Shen 2020; approval number: 001) and The First Hospital of Quanzhou Affiliated to Fujian Medical University (Quan Yi Lun 2020; approval number: 124). The requirement for written informed consent was not required for these two ethics committee owing to the retrospective nature of the study. Research was conducted in accordance with the relevant guidelines and regulations of the Chinese Health Commission on the prevention and control of COVID-19.

### Patients and setting

We conducted a retrospective cohort study of the clinical characteristics of 47 patients with COVID-19 who were admitted to three designated hospitals in Quanzhou City, on the southeast coast of Fujian Province, China, between January 21, 2020 and March 6, 2020. Since the first COVID-19 case was confirmed on January 23, 2020, 22 cases of COVID-19 were diagnosed in just 1 week, and 47 cases were all confirmed within 3 weeks in Quanzhou. The criteria for all confirmed cases and their discharge conformed to the *Diagnosis and Treatment Plan for Novel Coronavirus-Infected Pneumonia (6th Edition)*^14^. The discharge criteria were: normal temperature for at least 3 days; significantly improved respiratory symptoms; pulmonary imaging showing significant improvement in acute exudative lesions; and nucleic acid test of two consecutive respiratory specimens, sampled at least 1 day apart, which was negative.

We classified 47 patients into the control group (length of hospital stay, < 21 days) and the delayed discharge group (length of hospital stay, ≥ 21 days). All patients underwent at least two SARS-CoV-2 nucleic acid tests. The specimens included throat swabs, sputum, nasopharyngeal swabs, alveolar lavage fluid, feces, anal swabs, and urine samples. The SARS-CoV-2 nucleic acid test results were judged based on real-time quantitative polymerase chain reaction. The time to SARS-CoV-2 nucleic acid-negative conversion refers to the time from the onset of symptoms to the date of the first negative nucleic acid test result of at least two consecutive negative test results. Negative results followed by a positive result were considered false-negative.

### Data collection

Data on patient demographics, symptoms, signs, complications, laboratory test results, lung computed tomography findings, and clinical treatment were collected from the inpatient record system. Two independent reviewers extracted data and evaluated the suitability of the raw data. Before the final analysis, all differences were resolved through discussion.

### Statistical analyses

All statistical analyses were conducted using SPSS 25.0 (IBM Corp., Armonk, NY, USA). Figures were plotted using GraphPad Prism 8.1 (GraphPad Software Inc., San Diego, CA, USA). Continuous variables were compared using *t*-tests or Mann–Whitney *U* test and expressed as the median and (IQR). Categorical variables were compared using χ^2^ tests or Fisher’s exact test and expressed as frequencies and percentages. Variables showing significant differences were further analyzed using univariate and multivariate logistic regression to identify factors leading to delayed discharge of patients. *P* values < 0.05 were considered statistically significant.

## Results

### Baseline characteristics

In total, 47 patients were enrolled in this study, including 27 in the delayed discharge group and 20 in the control group. The median length of hospital stay for all patients was 22 days. The median age of patients in the delayed discharge group (41 [range 31–54] years) was higher than that of patients in the control group (35 [range 31–45] years); however, the difference was not significant (*P* = 0.13). Table 1 shows the clinical characteristics according to group. No significant differences were observed between the groups in the prevalence of comorbidities, including hypertension, diabetes, cerebrovascular disease, malignant tumors, liver disease, and chronic respiratory disease, surgical history, and the incidence of fever, cough, lassitude, headache, hemoptysis, chest tightness, shortness of breath, rhinorrhea, dry throat, and nausea and vomiting. However, the number of patients with diarrhea and anorexia was significantly higher in the delayed discharge group than in the control group (both, *P* = 0.03). Similarly, no significant differences were observed between the groups in the time from symptom onset to admission, partial pressure of oxygen on admission, oxygenation index, mean arterial pressure, heart rate, and respiration.

**Table 1:** Demographic and epidemiological characteristics of patients with COVID-19.

	All patients (n = 47)	Hospitalization days < 21 (n = 20)	Hospitalization days ≥ 21 (n = 27)	*P* value
Age, median (IQR), years	38 (31–50)	35 (31–45)	41 (31–54)	0.13
BMI, Median (IQR) (kg/m^2^)	23.9 (20.7–26.4) (n = 39)	23.4 (21.7–25.8) (n = 19)	24.1 (20.1–28.3) (n = 20)	0.77
< 18.5	3 (7.69)	2 (10.53)	1 (5.00)	0.79
≥ 23	23 (58.97)	12 (63.16)	11 (55.00)	0.19
Sex, male	24 (51.06)	8 (40.00)	16 (59.26)	0.19
Exposure of seafood market in South China	1 (2.13)	1 (5.00)	0	0.43
Live in Wuhan ≥ 2 weeks	34 (72.34)	15 (75.00)	19 (70.37)	0.73
**Complications**				
Hypertension	10 (21.28)	3 (15.00)	7 (25.93)	0.59
Diabetes	5 (10.64)	3 (15.00)	2 (7.41)	0.72
Cerebrovascular disease	1 (2.13)	0	1 (3.70)	> 0.99
Malignant tumor	1 (2.13)	1 (5.00)	0	0.43
Chronic liver disease	11 (23.40)	6 (30.00)	5 (18.52)	0.57
Respiratory disease	4 (8.51)	1 (5.00)	3 (11.11)	0.83
Previous surgery	9 (19.15)	4 (20.00)	5 (18.52)	> 0.99
**Signs and symptoms**				
Fever	39 (82.98)	15 (75.00)	24 (88.89)	0.39
Dry cough	11 (23.40)	3 (15.00)	8 (29.63)	0.41
Expectoration	26 (55.32)	13 (65.00)	13 (48.15)	0.25
Hemoptysis	4 (8.51)	1 (5.00)	3 (11.11)	0.83
Fatigue	19 (40.43)	6 (30.00)	13 (48.15)	0.21
Anorexia	6 (12.77)	0	6 (22.22)	0.03
Headache	8 (17.02)	4 (20.00)	4 (14.81)	0.94
Diarrhea	10 (21.28)	1 (5.00)	9 (33.33)	0.03
Pharyngalgia	13 (27.66)	6 (30.00)	7 (25.93)	0.76
Shortness of breath	9 (19.15)	4 (20.00)	5 (18.52)	> 0.99
Chest tightness/pain	11 (23.40)	5 (25.00)	6 (22.22)	> 0.99
Stuffy and runny nose	7 (14.89)	3 (15.00)	4 (14.81)	> 0.99
Nausea and vomiting	2 (4.26)	0	2 (7.41)	0.50
Time from onset to admission (Quartile interval, day)	3 (1–6) (n = 46)	3 (2–6)	2 (1–5) (n = 26)	0.35
Partial oxygen pressure (quartile interval, mmHg)	90.7 (79.3–104) (n = 36)	92.2 (80.1–106) (n = 15)	89.1 (77.1–103) (n = 21)	0.29
Oxygenation index (quartile interval)	431.5 (366.3–494.8) (n = 36)	439 (382–505) (n = 15)	424 (358–490) (n = 21)	0.29
Mean arterial pressure (quartile interval, mmHg)	97 (87–104) (n = 46)	93 (87–103) (n = 20)	97 (89–102) (n = 26)	0.28
Heart rate (per minute)	89 (85–96)	89 (86–99)	89 (84–92)	0.21
Respiratory rate (per minute)	20 (20–21)	20 (20–20)	20 (20–22)	0.65

Normal BMI (Asia standard): 18.5–22.9 kg/m^2^.

*BMI* body mass index, *IQR* interquartile range.

### Laboratory tests

**Figure 1:**
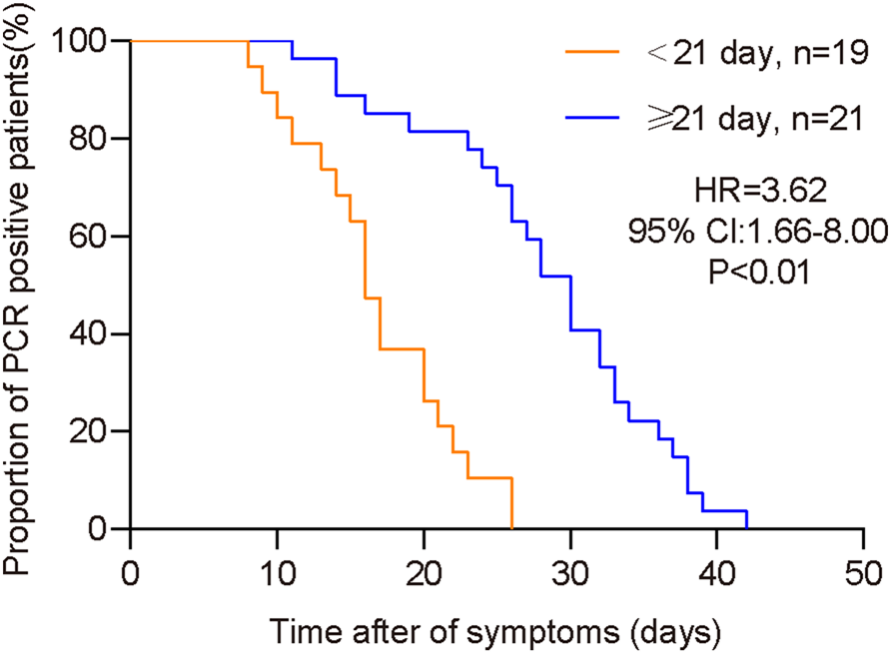
Time to severe acute respiratory syndrome coronavirus (SARS-CoV-2) nucleic acid-negative conversion by PCR analysis of upper respiratory tract samples. *CI* confidence interval, *HR* hazard ratio, *PCR* polymerase chain reaction.

Table 2 shows the laboratory test results according to group. The proportion of patients with decreased white blood cell counts, C-reactive protein and complement C3 levels, and the time to SARS-CoV-2 nucleic acid-negative conversion was significantly higher in the delayed discharge group than in the control group (*P* = 0.03, 0.01, 0.04, and < 0.01, respectively). The most significant difference was observed in the time to SARS-CoV-2 nucleic acid-negative conversion (*P* < 0.01), with the shortest and longest time to negative conversion being 8 and 42 days, respectively (Fig. 1). No significant difference in lymphocyte counts, neutrophil counts, monocyte counts, neutrophil/lymphocyte ratios, procalcitonin levels, D-dimer levels, lactate dehydrogenase levels, hepatic function (total bilirubin, albumin, aspartate aminotransferase, and alanine aminotransferase levels), renal function (blood urea nitrogen and creatinine levels), cardiac enzymes (troponin levels and creatinine kinase-myocardial band), or humoral immunity (immunoglobulin A, immunoglobulin G, immunoglobulin M, and complement C3 levels) was observed between the two groups. Regarding lung computed tomography manifestations, no significant difference was observed between the groups in the presence of bilateral changes or ground-glass opacities; however, air bronchograms were significantly more common in the delayed discharge group than in the control group (18/27 [67%] vs. 4/20 [20%]; *P* = 0.004).

**Table 2:** Laboratory findings of patients with COVID-19.

	All patients (n = 47)	Hospitalization days < 21 (n = 20)	Hospitalization days ≥ 21 (n = 27)	*P* value
**Laboratory findings**				
Leucocytes (*10^9/L)	5.47 (4.40–6.48)	5.43 (4.40–6.35)	5.75 (3.81–6.48)	0.29
< 3.5 (%)	6 (12.77)	0	6 (22.22)	0.03
Neutrophil (*10^9/L)	3.23 (2.43–3.87)	3.22 (2.46–3.81)	3.41 (2.43–4.06)	0.31
Lymphocytes (*10^9/L)	1.55 (1.10–1.94)	1.56 (1.12–1.90)	1.44 (1.03–1.88)	0.57
< 1.1 (%)	12 (25.53)	3 (15.00)	9 (33.33)	0.28
Neutrophil-to-Lymphocyte ratio	2.10 (1.56–2.92)	1.83 (1.53–3.37)	2.37 (1.63–2.83)	0.78
Monocytes (*10^9/L)	0.48 (0.36–0.62)	0.44 (0.34–0.54)	0.48 (0.38–0.65)	0.76
Platelet (*10^9/L)	228 (190–265)	236 (203–262)	212 (187–269)	0.66
Erythrocyte sedimentation rate (mm/h)	17 (11–22) (n = 10)	18 (16–19) (n = 2)	17 (9–30) (n = 8)	> 0.99
Prothrombin time (S)	11.5 (11–11.8) (n = 43)	11.4 (11–11.6) (n = 19)	11.6 (11–11.8) (n = 24)	0.71
> 13 (%)	2 (4.26)	1 (5.00)	1 (3.70)	> 0.99
D-dimer (mg/L)	0.32 (0.26–0.47)	0.38 (0.28–0.52)	0.29 (0.25–0.36)	0.13
Creatine kinase (U/L)	69 (44–103)	58 (42–93)	72 (52–108)	0.57
Creatine kinase isoenzyme (U/L)	14 (10–17)	11 (8–15)	15 (12–18)	0.57
Lactic dehydrogenase (U/L)	172 (149–203)	159 (144–188)	176 (156–208)	0.41
Albumin (g/L)	39.2 (36.3–42.1)	38.1 (35.9–41.8)	39.4 (37.1–42.5)	0.68
Albumin -to-Globulin ratio		1.4 (1.3–1.5)	1.3 (1.1–1.5)	0.65
< 1.2 (%)	12 (25.53)	3 (15.00)	9 (33.33)	0.28
Alanine transaminase (U/L)	23 (14–34)	24 (12–35)	23 (15–28)	0.63
> 40 (%)	8 (17.02)	2 (10.00)	6 (22.22)	0.48
Aspartate aminotransferase (U/L)	24 (19–30)	20 (16–26)	27 (21–32)	0.13
> 35 (%)	5 (10.64)	1 (5.00)	4 (14.81)	0.55
Total bilirubin (μmol/L)	15.9 (11.3–23.9)	17.8 (9–24.2)	15.5 (11.7–21.0)	0.78
Blood urea nitrogen (mmol/L)	3.56 (2.93–4.08)	3.58 (2.88–4.04)	3.56 (3.05–4.08)	0.74
Creatinine (μmol/L)	64.6 (53.9–77.6)	57 (51.1–70.9)	68.7 (57.1–80.2)	0.15
Troponin I (ng/ml)	0.002 (0.001–0.003) (n = 39)	0.001 (0.001–0.003) (n = 15)	0.003 (0.001–0.003) (n = 24)	0.33
Procalcitonin > 0.1 ng/ml (percentage, %)	2 (4.88) (n = 41)	2 (10.00)	0	0.18
C-reactive protein (mg/L)	3.65 (0.51–14.4)	0.52 (0.49–4.09)	6.33 (3.42–18.9)	0.01
Complement C3	0.81 (0.74–0.95) (n = 45)	0.78 (0.69–0.88) (n = 19)	0.85 (0.75–1.08) (n = 26)	0.04
Complement C4	0.2 (0.15–0.3) (n = 45)	0.18 (0.15–0.22) (n = 19)	0.23 (0.18–0.36) (n = 26)	0.09
Negative conversion time of SARS-CoV-2 RNA after onset of symptoms	24 (16–30)	16 (14–21)	30 (25–34)	< 0.001
Positive for influenza A/B/RS virus IgM	14 (29.79)	4 (20.00)	10 (37.04)	0.35
**Imaging features**				
Flaky/patchy shadows	39 (82.98)	14 (70.00)	25 (92.59)	0.10
Ground glass shadow	29 (61.70)	13 (65.00)	16 (59.26)	0.69
Broncho meteorology	22 (46.81)	4 (20.00)	18 (66.67)	< 0.01
Halo/anti-halo sign	5 (10.64)	1 (5.00)	4 (14.81)	0.55
Consolidation	12 (25.53)	2 (10.00)	10 (37.04)	0.08
Peripheral tape, subpleural	30 (63.83)	10 (50.00)	20 (74.07)	0.09

### Treatment

Table 3 shows the treatment provided according to group. The proportion of patients who were treated with traditional Chinese medicine and thymalfasin was significantly higher in the delayed discharge group than in the control group (both, *P* < 0.01). However, no significant difference was observed between the groups in the proportion of patients who received corticosteroids, antivirals, antibiotics, probiotics, acetylcysteine tablets, ambroxol/aminophylline, and supplemental oxygen or assisted ventilation.

**Table 3:** Treatment of patients with COVID-19.

	All patients (n = 47)	Hospitalization days < 21(n = 20)	Hospitalization days ≥ 21(n = 27)	*P* value
Antiviral therapy	47 (100)	20 (100)	27 (100)	> 0.99
Antibiotic	16 (34.04)	6 (30.00)	10 (37.04)	0.62
Glucocorticoid	13 (27.66)	3 (15.00)	12 (44.44)	0.06
Traditional Chinese medicine	36 (76.60)	9 (45.00)	27 (100)	< 0.001
Intestinal probiotics	42 (89.36)	19 (95.00)	23 (85.19)	0.55
Acetylcysteine	39 (82.98)	14 (70.00)	25 (92.59)	0.10
Ambroxol/theophylline	8 (17.02)	3 (15.00)	5 (18.82)	> 0.99
Thymalfasin	19 (40.43)	3 (15.00)	16 (59.26)	< 0.01
Oxygen uptake	20 (42.55)	7 (35.00)	13 (48.15)	0.37
High flow oxygen therapy	2 (4.26)	1 (5.00)	1 (3.70)	> 0.99
Noninvasive ventilator	1 (2.13)	1 (5.00)	0	0.43
Invasive ventilation	1 (2.13)	0	1 (3.70)	> 0.99
Ventilation treatment in prone position	1 (2.13)	0	1 (3.70)	> 0.99

### Univariate and multivariate logistic regression analyses

**Figure 2:**
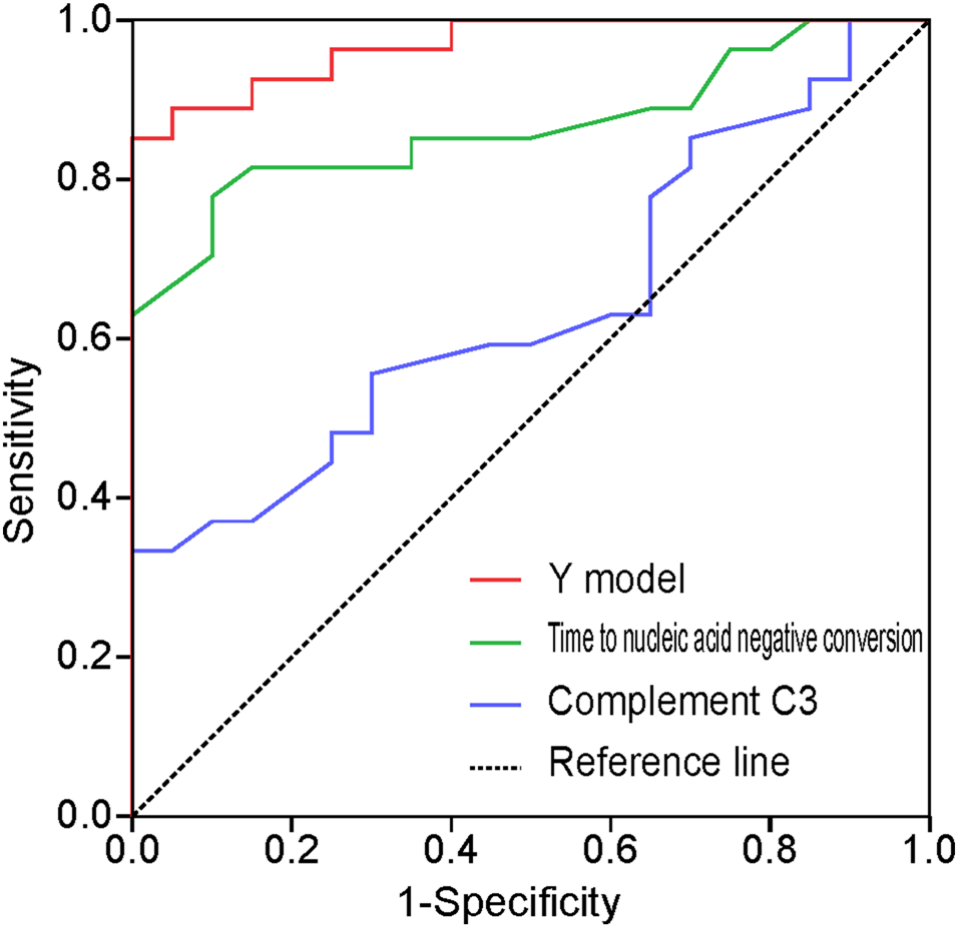
Receiver operating characteristic curves of the Y model, complement C3 levels, and time to nucleic acid-negative conversion for predicting delayed discharge from hospital.

Based on the above results, we performed univariate logistic regression analysis of nine factors, namely, diarrhea, anorexia, white blood cell counts, complement C3 levels, C-reactive protein levels, air bronchograms, the time to SARS-CoV-2 nucleic acid-negative conversion, treatment with traditional Chinese medicine, and thymalfasin treatment. Results of the logistic regression analyses are shown in Table 4. Diarrhea, thymalfasin treatment, complement C3 levels, the time to SARS-CoV-2 nucleic acid-negative conversion, and air bronchograms were associated with prolonged hospital stay. Multivariate logistic regression analysis showed that only complement C3 levels and the time to SARS-CoV-2 nucleic acid-negative conversion were independently associated with delayed discharge from hospital (Table 4). According to their regression coefficients, a binary logistic regression model was established as follows: Y = 0.13 × complement C3 + 0.39 × the time to nucleic acid-negative conversion − 16.94. Receiver operating characteristic curve analysis was used to evaluate the diagnostic value of this model. The area under the curve for predicting delayed discharge from hospital was 0.97 (95% confidence interval [CI] 0.93–1.00), which was significantly higher than that of complement C3 levels (0.64, 95% CI 0.49–0.80, *P* < 0.001) and the time to SARS-CoV-2 nucleic acid-negative conversion (0.87, 95% CI 0.76–0.97, *P* < 0.01). With a cut-off value of 0.52, the sensitivity, specificity, and positive and negative predictive values of this model were 89%, 95%, 95%, and 91%, respectively (Fig. 2).

**Table 4:** Univariate and multivariate logistic regression analysis of factors associated with delayed discharge of patients with COVID-19.

	Univariate		Multivariate	
	OR (95% CI)	*P* value	OR (95% CI)	*P* value
Diarrhea	0.11 (0.01–0.92)	0.04		
Thymalfasin	0.07 (0.01–0.34)	0.001		
Complement C3	1.04 (1.00–1.08)	0.04	1.14 (1.02–1.27)	0.03
Broncho meteorology	8 (2.06–31.07)	0.003		
Negative conversion time of SARS-CoV-2 RNA	1.24 (1.10–1.39)	< 0.001	1.48 (1.09–2.03)	0.01

*CI* confidence interval, *OR* odds ratio.

## Discussion

Previous research on COVID-19 has characterized the epidemiology, clinical features, and imaging findings of patients with COVID-19. However, information on risk factors for delayed discharge from hospital is limited. In this study, we identified risk factors associated with delayed discharge among 47 patients with COVID-19 who were admitted to three designated hospitals for COVID-19 in Quanzhou City, Fujian Province, China.

A previous study showed that the average length of hospital stay for patients with COVID-19 was 24.7 days^15^. Therefore, we selected 21 days (3 weeks) as the threshold for delayed discharge, which is easy to recall. In our study, the median (interquartile range [IQR]) length of hospital stay for patients with mild, moderate, and severe or critical disease was 20 (15–24), 22 (16–30), and 29 (22–35) days, respectively, showing a gradually increasing trend. Univariate and multivariate logistic regression analyses both showed that complement C3 levels and the time to SARS-CoV-2 nucleic acid-negative conversion were independent predictors of delayed discharge from hospital. This result is different from that reported in a previous study, which showed that patients with low arterial pressure of oxygen/fraction of inspired oxygen ratios, low lymphocyte counts, severe clinical presentations, and corticosteroid treatment are more likely to experience delayed clinical recovery^16^. This may be related to the relatively mild illness and small number of cases in our region. Moreover, we constructed a non-invasive Y model, including these variables, to predict delayed discharge from hospital. The sensitivity and specificity of this model were 89% and 95%, respectively.

Complement C3 levels were independently associated with delayed discharge from hospital. SARS-CoV-2 infection can activate innate and adaptive immune responses; however, uncontrolled innate inflammatory response and impaired adaptive immune response may lead to harmful local and systemic tissue injuries^17^. Complement C3, an immune inflammatory mediator, participates in the immune response of the body to eliminate viruses or bacteria. Increased complement C3 levels indicate that the body’s complement system has been activated, and there may be a strong inflammatory reaction. The stronger the inflammatory reaction, the greater the likelihood of severe disease and admission to an intensive care unit^18^. If a complement inhibitor is administered in the early stages of infection, the inflammatory injury can be alleviated, facilitating recovery^17^.

Negative SARS-CoV-2 nucleic acid test results indicate that the virus has been cleared, which is an important indicator of disease prognosis. Xiao et al*.*^19^ observed that the majority of patients who were positive for SARS-CoV-2 within 3 weeks after the onset of symptoms subsequently gradually became negative until they all tested negative at 6 weeks, suggesting SARS-CoV-2 viral replication has a relatively long period. In this study, the median time to SARS-CoV-2 nucleic acid-negative conversion was 24 days, consistent with the results of the study by Xiao et al*.*^19^; however, this was significantly longer than those obtained by Chen et al*.*^20^, which might be due to the longer time interval between SARS-CoV-2 nucleic acid tests in this study. In future diagnosis and treatment, in patients with improved clinical symptoms and imaging findings, the interval between SARS-CoV-2 nucleic acid tests should be reduced to shorten the hospital stay and reduce the financial burden of patients.

Although there have been no previous reports of diarrhea being associated with delayed discharge from hospital, a study reported a confirmed case with diarrhea as the first symptom, and patients with diarrhea had a higher probability of requiring ventilator support and being admitted to the intensive care unit^21^. In this study, only one critically ill patient required tracheotomy and ventilator support. Similarly, this patient had diarrhea, indicating that patients with COVID-19 with diarrhea need ventilator support and intensive care more often than those without diarrhea^22^. The presence of gastrointestinal symptoms is associated with a higher risk of hospitalization, which becomes more pronounced as the severity of the disease increases^23,24^. Furthermore, 90% (9/10) of patients with diarrhea in this study were in the delayed discharge group, implying that the length of hospital stay was longer for patients with diarrhea than for those without diarrhea; however, this finding requires further verification in multicenter studies with a larger sample size. Although the pathogenesis of gastrointestinal symptoms in patients with COVID-19 remains unclear, it may be related to the recruitment of inflammatory cells into the gastrointestinal tract, which releases inflammatory factors, and the expression of angiotensin-converting enzyme 2 in the gastrointestinal tract^25^. Similarly, studies have shown that air bronchograms are common during disease progression^26^ and are more common in fatal cases than in survivors, indicating its predictive value. Although univariate logistic regression analysis in this study equally showed that diarrhea and air bronchograms were associated with delayed discharge from hospital, the multivariate logistic regression analysis showed that they were not associated with delayed discharge. The lack of a statistically significant association may be attributable to the small sample size of this study.

Previous research reported that glucocorticoid use in patients with COVID-19 may lead to prolonged hospital stay^27^, and a study suggested that high-dose glucocorticoid use leads to an increased risk of mortality in patients with COVID-19^28^. In this study, although we treated patients on the short-term with low-dose glucocorticoid therapy, those who received glucocorticoid therapy tended to stay longer in hospital (*P* = 0.06). Therefore, the necessity of glucocorticoid use requires further investigation.

Other research has shown that male sex, delayed admission after the onset of symptoms, and invasive mechanical ventilation are factors associated with longer time to SARS-CoV-2 RNA-negative conversion^29^; however, they were not associated with delayed discharge in this study. A possible explanation is that SARS-CoV-2 RNA-negative conversion is only one of the discharge criteria, and temperature, clinical symptoms, and pulmonary imaging results were similarly considered.

This study had some limitations. First, the number of cases in this region is relatively small, and a larger sample size may be required for verification. Second, the antiviral therapy included lopinavir/ritonavir and interferon-α. Since nearly all patients were administered this antiviral therapy, we could not judge the effect of this treatment on delayed discharge. Third, the immune system is closely related to the severity of COVID-19 and clinical outcomes^30,31^, which may affect the length of hospital stay; however, we did not analysis this in our study.

In conclusion, high complement C3 levels and an extended time to SARS-CoV-2 RNA-negative conversion are risk factors for delayed discharge from hospital. Therefore, repeated and continuous monitoring of complement C3 levels and nucleic acid load are helpful for early assessment of discharge indications to increase the availability of beds and enable more patients to be treated in hospital.
